# Efficacy of Transcutaneous Electric Nerve Stimulation (TENS) on Whole Salivary Flow Rate: A Descriptive Observational Study

**DOI:** 10.30476/DENTJODS.2021.89773.1441

**Published:** 2022-06

**Authors:** Sunbul Tabrez, Neelkant Patil, Mohit Sareen, Manoj Meena, Nitesh Tyagi, Shobhit Kaswan

**Affiliations:** 1 Postgraduate Student, Oral Medicine and Radiology at Rajasthan Dental College and Hospital, Jaipur, Rajasthan, India; 2 Dept. of Oral Medicine and Radiology, Rajasthan Dental College & Hospital, Jaipur, Rajasthan, India

**Keywords:** Transcutaneous electric nerve stimulation (TENS), Salivary gland, Diabetes mellitus, Hyposalivation

## Abstract

**Statement of the Problem:**

Saliva is a precious oral fluid that contributes to oral health and when its quantity is diminished, it hampers the quality of life. Individuals suffering from diabetes have a complaint of reduced salivation due to the consumption of xerogenic drugs and autonomic neuropathy.

**Purpose:**

Our study aims to evaluate the effectiveness of the transcutaneous electric nerve stimulation (TENS) device on the salivary flow rate with respect to age and gender in Jaipur population.

**Materials and Method:**

A descriptive observational study was carried out on individuals in Jaipur at the Department of Oral Medicine and Radiology at Rajasthan Dental College and Hospital during a period of 7 months. The study consisted of 200 individuals who were divided into two groups. Unstimulated and stimulated saliva were collected for 5 minutes in a graduated beaker. Stimulated saliva was collected after keeping the TENS unit activated at 50Hz. Kolmogorov-Smirnov, Shapiro-Wilks normality tests and Mann Whitney U test were done. The *p* value <0.05 was considered to be significant.

**Results:**

The TENS unit was effective in increasing the quantity of stimulated saliva and a highly statistical significance was seen in age groups. TENS was also found to be more effective in increasing saliva in diabetic individuals. The mean unstimulated salivary rate was 1.64ml/5min and the mean stimulated salivary rate was 1.914ml/5min for Group I. The mean unstimulated salivary rate was 1.231ml/5 min and the mean stimulated salivary rate was 1.547ml/5 min for Group II. The p value for Group I and II for unstimulated saliva was 0.01 and for stimulated saliva was 0.03.

**Conclusion:**

It seems that TENS has shown positive results in increasing salivary secretions and salivary values may diminish with age; therefore, TENS might be used in aged individuals as well as in diabetic patients to increase the quantity of saliva.

## Introduction

Saliva is a precious oral fluid that is often taken for granted but we need to realize that it is very critical for preserving and maintaining oral health [ 1 ]. Saliva has innumerable functions in the mouth that include lubrication, protection of the oral mucosa, pellicle formation, digestion, removal of food debris and has healing properties. The proteins present in the saliva have anti-viral, anti-bacterial, and anti-fungal properties [ 2 ]. Saliva in its normal quantity and composition acts as the most important aspect of the host immune system by protecting the oral cavity from various diseases [ 3 ]. Unstimulated saliva is a mixture of secretions, which enter the mouth in the absence of exogenous stimulus, whereas, stimulated saliva refers to the saliva that is secreted in response to either masticatory or gustatory stimulations [ 4 ]. Stimulated salivary flow rates drastically change the percentage of contributions from each gland, with the parotid contributing to more than 50% of the total amount of salivary secretions [ 5 ].

Hyposalivation has been found to be associated with diabetes mellitus [ 6 ]. Xerostomia in type I diabetes mellitus could be dependent on glucose control, whereas in type 2 diabetes mellitus, salivary secretion seems to be pre-disposed by both xerogenic drugs and autonomic neuropathy [ 7 ]. 

Application of electric impulses to one or more of the three components of the salivary reflex arch should theoretically improve salivary secretion and lessen the various long-term effects of hyposalivation [ 8 ].

Transcutaneous electric nerve stimulation (TENS) is a simple, inexpensive, and non-invasive modality that uses electric current to activate nerves for therapeutic reasons. It is a non-pharmacological method of pain management for which it is widely used [ 9 ].

Earlier, TENS was majorly concentrated on pain, but few clinical trials have been conducted to identify the effect of electrical nerve stimulation on salivary flow [ 8 ]. As the controversy between the articles existed, this study was done to determine the efficacy of TENS on salivary flow rate with two methods (stimulated and unstimulated saliva) in relation to both gender and age including healthy adult, non-diabetic, as well as diabetic individuals since diabetes is one of the diseases that affect salivary secretions leading to hyposalivation.

## Materials and Method

This descriptive type of observational study was conducted at the Department of Oral Medicine and Radiology at Rajasthan Dental College and Hospital during a period of 7 months (August 2019- February 2020). The ethical certificate was obtained from the college (RDCH /Ethical/2019/708).

The total sample size of the study was decided to be200 participants after a discussion with the statistician.

Out of the total 200 individuals, 100 healthy individuals (50males and 50 females) within the age group of 20-40 years were included in Group I. Group II consisted of 100 individuals of age >40 years, out of which 50 were non-diabetics (25 males and 25 females) and the remaining 50 were diabetic individuals (25 males and 25 females). Patients who had any deleterious habits like alcohol or drug consumption and tobacco in any forms, usage of any medications, history of salivary gland pathology that may affect salivary flow, pregnancy, history of head and neck radiotherapy, granulomatous diseases, Sjögren’s syndrome and any systemic diseases except diabetes were excluded from the study as most of these lead to hyposalivation and we wanted our study to be exclusive to notice changes in salivary values due to diabetes alone. 

**Figure 1:**
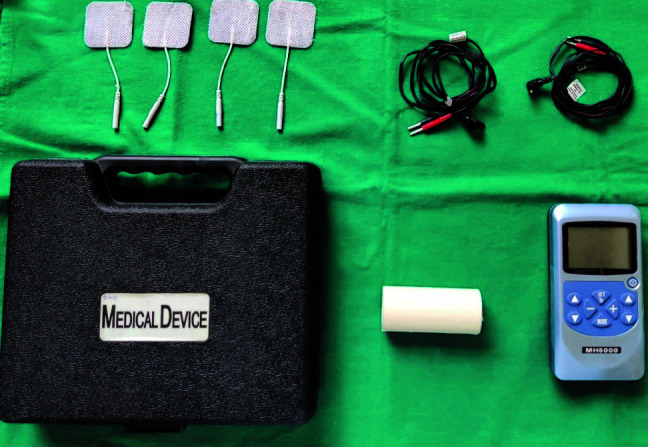
The transcutaneous electric nerve stimulation (TENS) device used for the study

**Figure 2:**
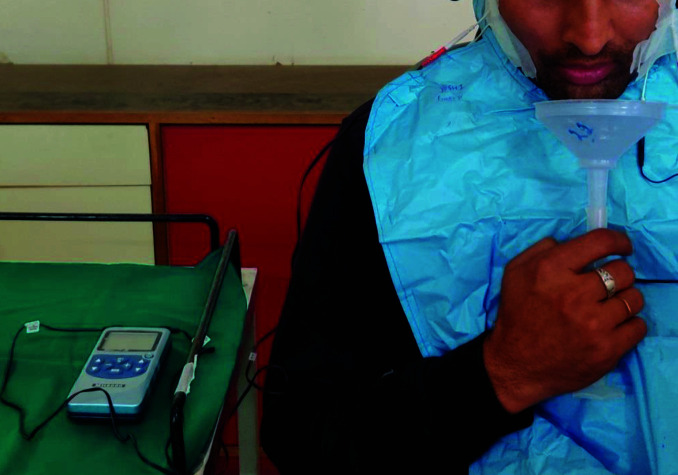
Saliva collection by “draining” method

Patients were explained about the procedure and informed consents were taken. For the diabetic individuals the random blood sugar (RBS) levels were obtained first by using a tabletop glucometer (Dr. Morepen GlucoOne blood glucose monitoring system BG 03) and only those individuals who were under medication for at least one year were included. The patients were seated comfortably on the dental chair and were asked to refrain from eating or drinking 1 hour prior to saliva collection. The procedure was done during the early morning hours between 9 am to 12 am. Unstimulated saliva was collected for 5 minutes in a graduated beaker by draining method [ 3 ]. The readings were noted after the bubbles at the top of the saliva levels settled. For the collection of stimulated saliva, the skin overlying the parotid gland were cleaned with an alcohol swab, then the electrode pads were placed on the overlying skin and secured via the help of micropore. A dual channel TENS unit (Medihightech Med CO.LTD, Taiwan; MH8000) with voltage of 9V, intensity of 0-80mA, pulse rate 2 Hz-20Hz in 1Hz/step, pulse width 10 µsec/ step, contraction time and relaxation time of 2-90 seconds was used for the study (Figure 1). It was activated at 50 Hz and the optimal intensity was obtained by asking the patient to raise his hand on reaching the maximum threshold of the patient. Stimulated saliva after the activation of TENS was collected for five minutes by the same way of draining method into the graduated beakers and their readings were noted (Figure 2). 

The data collected were analyzed by SPSS software, version 20.0. The *p* Value of < 0.05 was set as statistical significance. Kolmogorov-Smirnov and Shapiro-Wilks normality tests were used to check whether the variables follow normal distribution. For variables that did not follow normal distribution, Mann Whitney U test was used to compare mean between the groups.

## Results

**Figure 3:**
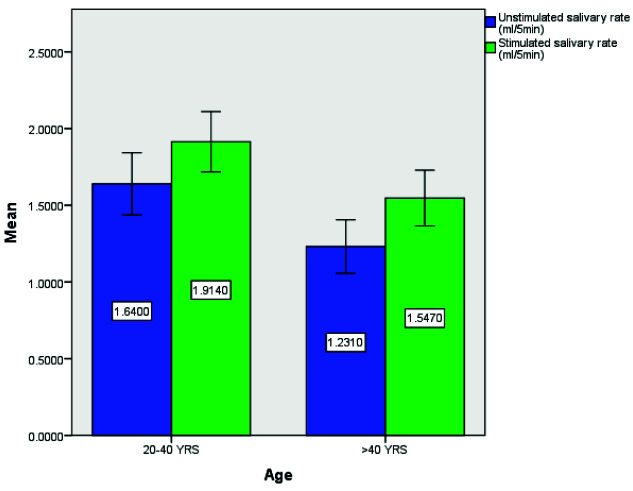
Graphical distribution of salivary production by the effect of transcutaneous electric nerve stimulation (TENS) on different age groups

The mean age for Group I was 28.36±3.1, for Group II non-diabetic individuals was 53.56±7.8 and for Group II diabetic individuals was 52.16±7.4. 85 out of 100 individuals experienced an increase in stimulated saliva, 9 had no change and 6 had a decrease in Group I (20-40years), In Group II (>40years) 83 patients out of 100 patients had an increase in stimulated saliva, 9 had a decrease and 8 patients had no change in salivary flow. Individuals who did not show any change were the ones who showed no flow initially. Only those individuals who had an initial saliva flow already were the ones who showed a significant increase. For age group 20-40 years, the mean unstimulated salivary rate was 1.64ml/5min and the mean stimulated salivary rate was 1.914ml/5min. In age >40years, the mean unstimulated salivary rate was 1.231ml/5min and the mean stimulated salivary rate was 1.547ml/5min. Mann Whitney U test was applied; the p value for both Group I (20-40 years) and Group II (>40 years) for unstimulated salivary flow was 0.01 and for stimulated salivary flow was 0.03, respectively. A significant difference was seen between unstimulated and stimulated salivary flow while comparing different age groups (Figure 3).

**Figure 4:**
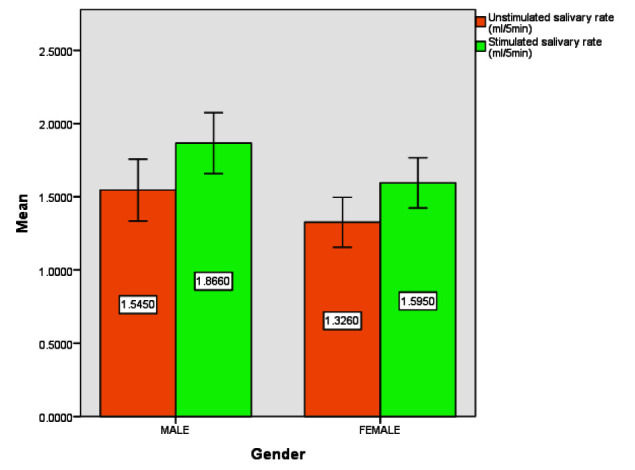
Graphical distribution of salivary production by the effect of transcutaneous electric nerve stimulation (TENS) on both males and females

For Group I (20-40years), the mean unstimulated salivary rate was 1.64ml/5min and the mean stimulated salivary rate was 1.914ml/5min. For Group II (>40years), the mean unstimulated salivary rate was 1.231ml/5min and the mean stimulated salivary rate was 1.547ml/ 5min. Mann Whitney U test was applied and the p value for unstimulated salivary production and for stimulated salivary production for both males and females was 0.198 and 0.083, respectively. There was no significant difference on salivary production considering gender when the effect of TENS on salivary production was done (Figure 4).

**Figure 5:**
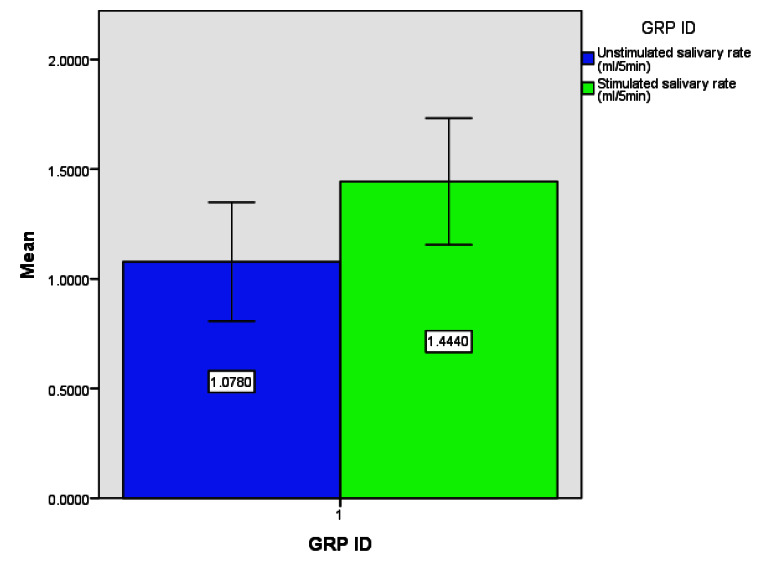
Graphical mean distribution of stimulated and unstimulated saliva on diabetic individuals by the effect of transcutaneous electric nerve stimulation (TENS)

**Figure 6:**
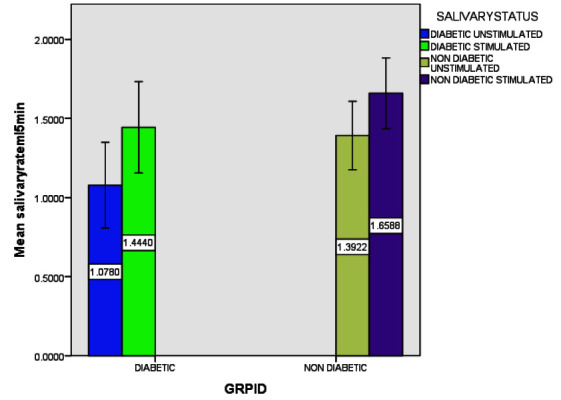
Comparative graphical distribution of mean and p value on non-diabetic and diabetic individuals by the effect of transcutaneous electric nerve stimulation (TENS)

For the diabetic individuals, the mean unstimulated salivary rate was 1.07ml/5min and the mean stimulated salivary rate was 1.44ml/5min. One-sample t-test was applied and the p value for unstimulated and for stimulated salivary production was 0.00; a significant difference was seen (Figure 5). The mean unstimulated salivary production for diabetic individuals was 1.07ml /5min and for non-diabetic individuals was 1.39ml/ 5min. Mann Whitney U test was applied and the *p* value for unstimulated salivary production for both diabetic and non-diabetic individuals was 0.002 and stimulated salivary production was 0.043; a significant difference was seen (Figure 6).

## Discussion

Reduced salivary secretion has been linked to diabetes and not many studies have been done regarding the use of electro-stimulation as a therapeutic modality in treating hypo-function of the salivary gland.

The present study showed that 168 participants of the study had an increase, which may be attributed to the mechanism by which TENS acts on the parotid glands. It causes stimulation of the auriculo-temporal nerve, which controls the secreto-motor drive of the parotid gland. On the application of the TENS device, parasympathetic supply gets stimulated and hence, produces watery and copious saliva. A total of 17 individuals had no change in saliva because these were those individuals who showed no flow initially. A total of 15 individuals had reduced saliva, which may be due to the fact that the brain may have perceived the electrical stimulus to be painful. In addition, for the diabetic individuals our study demonstrated that the mean unstimulated saliva was 1.08ml/5min, which increased to 1.44ml/5min after stimulation. Similar findings were noted in the study conducted by Dhillon *et al.* [ 10 ] where it was found that 87 out of the total 100 individuals demonstrated increase in saliva secretion, 3 had a decrease, and 10 experienced no change.Dhillon *et al.* [ 10 ] included 100 healthy individuals in their study and divided them into two groups, the first group having 50 individuals were of the age range of 20-40years and the second group consisting of 50 individuals with >60 years of age. Modified Carlson Crittenden cup was used and saliva was collected for five minutes. They reported that 43 individuals in the first group after TENS application and 44 individuals in the second group showed an increase in saliva secretion.

Similar to our studies, where an increase in stimulated saliva was seen, studies carried out by Bhasin *et al.* [ 11 ] reported that 96 out of 100 individuals responded positively to TENS therapy. Moreover, in the study conducted by Hargitai *et al.* [ 12 ], it was stated that 15 out of 22 healthy adult individuals also showed a significant increase in stimulated salivary flow.

 In the study by Manoj Kumar *et al.* [ 13 ], 62 out of 80 individuals showed an increase in saliva and the study conducted by Aggarwal *et al.* [ 14 ], 65 out of 80 individuals showed an increase in salivary flow rate after stimulation by TENS.

Bhasin *et al.* [ 11 ] conducted a study on 100 individuals of the age range of 20-69 years and they excluded individuals with systemic diseases and salivary gland disorders. They divided the participants into 5 groups of 20 each. TENS was activated for 5 minutes then after 30 minutes and after 24 hours. An increase in saliva was seen by 38.46% post-stimulation. 

Hargitai *et al.* [ 12 ] conducted the study on 22 healthy adult individuals with no salivary gland disorders. TENS was used for salivary stimulation and collected after 5minutes; two-thirds of individuals showed an increase post application of TENS. 

Aggarwal *et al.* [ 14 ] conducted the study on 80 healthy individuals, out of which 40 were male and 40 females. They were of the age range of 20-50 years Individuals having salivary gland pathologies, systemic diseases, and radiation therapy in the head and neck regions was excluded from their study. Saliva was collected by low forced spitting for 5minutes with and without TENS application and 65 individuals showed an increase in salivary stimulation.

In the present study, in the age group 20-40 years, the mean unstimulated saliva was 1.64ml/5min, which increased to 1.91ml/5min post-stimulation. This data was consistent with the study conducted by Bhasin *et al.* [ 11 ] and Singla *et al.* [ 15 ] where they also noticed an increase in saliva after stimulation by TENS. 

In this study, the individuals of the age range of 20-40 years had an increase in the both the mean unstimulated and stimulated salivary values which were 1.64ml/ 5min and 1.91ml/5min, respectively when compared to the individuals of the age >40 years who had mean unstimulated and stimulated salivary values of 1.23ml/ 5min and 1.54ml/5min, respectively. Our study correlates to findings of the study conducted by Manoj Kumar *et al.* [ 13 ] where maximum increase in salivary flow rate was seen in the age group of 20-29 years, which is 1.32±0.08 to 1.37±0.09. No statistical significance was seen between males and females in our study. In addition, in the study conducted by Dhillon M *et al.* [ 10 ], there was no statistical significance in terms of stimulated salivary flow in both genders as they had a limited number of female participants. 

The data in our study are also consistent with the studies conducted by Dyasnoor *et al.* [ 16 ], Singla *et al.* [ 15 ], Aggarwal *et al.* [ 14 ], and Singh *et al.* [ 17 ] where no significant data was found when comparing salivary production in relation to gender, but males produced more saliva than the females. Even after the application of TENS for salivary stimulation, males had increased saliva as compared to females. The reason for this could be that the females had a smaller salivary gland as compared to the males, hence, the amount of saliva produced was also lesser. In addition, there is a possibility of post-menopausal changes on the quantity of saliva in females.

Our study employed the draining method for saliva collection in which the saliva was collected passively in the floor mouth and was then allowed to drain into the graduated beaker without any forced self-stimulation. The studies conducted by Dhillon *et al.* [ 10 ], Aparna *et al.* [ 18 ] and Hargitai *et al.* [ 12 ] used the Carlson Crittenden cups for saliva collection which is considered to be a more accurate and reliable method of saliva collection. However, during their method, the mouth had to remain open passively so there could have been some amount of subjective drying of the mucosa while collection. Moreover, various studies conducted by Nimma *et al.* [ 19 ], Pattipati *et al.* [ 20 ], Aggarwal *et al.* [ 14 ], Singh *et al.* [ 17 ], Sakshi *et al.* [ 21 ], Lingam *et al.* [ 22 ], Manoj Kumar *et al.* [ 13 ], Mimansha *et al.* [ 23 ] and Singla *et al.* [ 15 ] used the low forced spitting method of saliva collection where certain amount of false increased salivation could have been possible.

In this study, the mean value of unstimulated saliva for the diabetics was 1.08ml/5min and for stimulated saliva was 1.44ml/5min. In the study conducted by Jagdhari *et al.* [ 24 ], where they included known cases of diabetic individuals as a part of their study, the mean values of unstimulated saliva were 2.53ml/5min and for stimulated saliva was 3.33ml/5min.

For the diabetic individuals, our study demonstrated that the mean unstimulated saliva was 1.08ml/5min, which increased to 1.44ml/5min. A significant change in saliva was seen post-stimulation. Dyasnoor *et al.* [ 16 ] conducted a similar study on diabetic individuals of the age range of 30-75years who had complaints of both xerostomia and hyposalivation. It was noted that there was significant increase of saliva when using TENS in continuous mode from the mean unstimulated saliva being 1.69ml/10mins, which increased to 1.88ml/10min post-stimulation. These findings are consistent with our study but in their study, the duration of saliva collection post-stimulation was set to 10 minutes since in 21-22% of the population, there is no parotid saliva production even when it is stimulated for five minutes

The limitation of our study was that the electrode pads were placed on the approximate location on the skin overlying the parotid gland and the exact anatomical measurements were not made. The diabetic staging of the individuals was not done and all individuals who were found to be diabetic were included in the study irrespective of the sugar levels. In addition, the patients could have been re-evaluated after 1 or 2 weeks after the application of the TENS device, to check for its effectiveness.

## Conclusion

It seems that TENS has shown positive results in increasing salivary secretions in different age groups and in the diabetic individuals. In the future, TENS might be used as an additional treatment modality to manage salivary gland dysfunctions.

## Conflict of Interest

The authors declare that they have no conflict of interest.
